# The impact of dynamic reversal potential on the evolution of action potential attributes during spike trains

**DOI:** 10.3389/fncom.2025.1740570

**Published:** 2026-01-09

**Authors:** Ahmed A. Aldohbeyb, Jozsef Vigh, Kevin L. Lear

**Affiliations:** 1 Department of Biomedical Technology, College of Applied Medical Sciences, King Saud University, Riyadh, Saudi Arabia; 2 School of Biomedical Engineering, Colorado State University, Fort Collins, CO, United States; 3 Department of Biomedical Sciences, Colorado State University, Fort Collins, CO, United States; 4 Department of Electrical and Computer Engineering, Colorado State University, Fort Collins, CO, United States

**Keywords:** action potential, cortical neurons, hippocampal neurons, Ion concentration, onset dynamics, onset rapidness, potassium dynamics, sodium dynamics

## Abstract

Action potentials (AP) are the basic elements of information processing in the nervous system. Understanding AP generation mechanisms is a critical step to understand how neurons encode information. However, an individual neuron might fire APs with various shapes even in response to the same stimulus, and the mechanisms responsible for this variability remain unclear. Therefore, we analyzed four AP attributes including AP rapidity and threshold during consecutive bursts from three neuron types using intracellular electrophysiological recordings. In response to consecutive current steps, the AP attributes in evoked spike trains show two distinctive patterns across different neurons: (1) The first APs from each train always have comparable properties regardless of the stimulus strength; (2) The attributes of the subsequent APs during each pulse monotonically change during the burst, where the magnitude of AP attribute change during each pulse increases with increasing stimulation strength. Various conductance-based models were explored to determine if they replicated the observed AP bursts. The observed patterns could not be replicated using the classical HH-type models, or modified HH model with cooperative Na^+^ gating. However, adding ion concentration dynamics to the model reproduced the AP attribute variation, and the magnitude of change during a pulse correlated with change in dynamic reversal potential (DRP), but failed to replicate the first AP attributes pattern. Then, the role of cooperative Na^+^ gating on neuronal firing dynamics was investigated. Inclusion of cooperative gating restored the first APs’ attributes and enhanced the magnitude of modeled variation of some AP attributes to better agree with observed data. We conclude that changes in local ion concentrations could be responsible for the monotonic change in APs attributes during neuronal bursts, and cooperative gating of Na^+^ channels can enhance the effect. Thus, the two mechanisms could contribute to the observed variability in neuronal response.

## 1 Introduction

Neuroscientists use neuron models to seek understanding of the complexity of neural dynamics. The choice from large variety of models requires trading off which phenomena to include to make the model realistic but still simple enough to implement (Brette, 2015). For this reason, a simple single-compartment model such as the Hodgkin and Huxley (HH) model (Hodgkin and Huxley, 1952), or even the simpler integrate-and-fire model (Brette, 2015), has been used for decades. Although growing evidence in central mammalian neurons indicates a discrepancy between experimental data and the HH model (Naundorf et al., 2006; Huang et al., 2012; Brette, 2013; Öz et al., 2015; Sato et al., 2018), one of which is the AP initiation attributes, most of the proposed improved models incorporate the basic functions and equations from the HH model (Meunier and Segev, 2002; Siciliano, 2012), and can successfully reproduce many of the general features of neuronal signals.

The HH model was based on several assumptions to simplify the mathematics, or due to the negligible impact of certain processes (Hodgkin and Huxley, 1952). The HH model simplifies neuronal complexity by assuming a uniform membrane potential within the studied compartment, which simplifies computation. Also, ion concentration changes during action potentials were considered to be insignificant, and hence were ignored, allowing the use of constant reversal potentials in the model. One the other hand, some assumptions were hypothesized due to the lack of direct evidence at the time the model was developed, when recording from a single ion channel was not yet possible. Thus, Hodgkin and Huxley suggested that each ion channel functions independently, with its activity solely influenced by changes in the membrane potential.

While neglecting ion concentration changes during neuronal firing is a reasonable approximation in invertebrate neurons, its validity in mammalian neurons is subject to debate (Ullah et al., 2009). Fluctuation in K^+^ and Na^+^ concentrations was noted in healthy cortical neurons (Singer and Dieter Lux, 1975; Amzica and Steriade, 2000; Felix et al., 2019; Perez et al., 2021) but more abundantly observed during abnormal activities such as seizure (Trevelyan et al., 2007; Gnatkovsky et al., 2008; Clapcote et al., 2009; Cressman et al., 2009; Igelström, 2013; Raimondo et al., 2015). Consequently, some computational models used to study neuronal abnormal activities have incorporated dynamical ion concentrations (Cressman et al., 2009; Krishnan and Bazhenov, 2011; Krishnan et al., 2015). However, ion concentration dynamics should be applied as well to understanding normal conditions (Singer and Dieter Lux, 1975; Amzica and Steriade, 2000). For example, the dynamic of Na^+^ concentration, in a theoretical study, was shown to have a huge impact on bursting activity in models of three neuron types (Zylbertal et al., 2017). Nonetheless, the vast majority of models incorporating dynamical ion concertation have been constructed to study pathological states, not normal neuronal behavior.

Our study here shows response variability to current-step stimulus, but the variability is more systematic than random. Analysis of electrophysiology recordings from public databases for three neuron types demonstrate that AP attributes including AP rapidity and threshold potential display recurring monotonic patterns in response to multiple current pulses. Further, the magnitude of change in these patterns during each pulse increases with increasing stimulus magnitude. Despite the stimulus-dependent evolution of AP attributes during a pulse, the attributes of the first spikes in each spike trains are strikingly similar, independent of stimulus strength. A variety of existing computational models were investigated, including a model with cooperative Na^+^ channel gating, but all failed to replicate the observed AP attribute patterns during spike trains. Thus, a novel conductance-based model that includes ion concentration dynamics and cooperative Na^+^ channels was constructed, which was found to replicate the experimentally observed spike patterns best. Changes in the dynamical reversal potential (DRP) due to changes in ion concentration was responsible for the evolution in AP attributes during stimulus pulses but also predicted monotonic changes in attributes of the first AP of each sequential pulse, contrary to the neural recordings. Notably, adding cooperative Na^+^ channels to the model equalized first-AP attributes and enhanced the magnitude of some AP attribute evolution during each stimulus pulse. With Na^+^ channel cooperativity, the modeled magnitude of intra-pulse variation in some AP attributes, such as rapidity, approximately agrees with experimental observations. Nonetheless, the improved model still underestimates the magnitude of systematic variation in other AP attributes, in particular, that in threshold potential. The impact of selected model parameters such as cell volume ratio were also investigated. Our study supports the hypotheses that ion concentration dynamics affect AP attributes during repetitive firing, and thus they may play a role in neuronal firing variability.

## 2 Materials and methods

### 2.1 AP attributes

The threshold voltage, amplitude, rapidity, and width of each recorded or simulated AP was analyzed using a MATLAB script. The AP threshold was measured as the membrane potential at which ${\dot{\text{V}}}_{\text{m}}$ exceeds 25 mV/ms. In some cases, the threshold potential was reported as the potential difference between the onset threshold of each spike and the threshold of the first AP (△_*thr*_), rather than the absolute value. The AP amplitude was measured from the threshold potential to the peak value, and the AP width was measured as the full width at half the AP amplitude. AP rapidity was measured using the inverse of the full width at half the maximum value of the rising phase of the membrane potential second time derivative (IFWd^2^) (Aldohbeyb et al., 2020, 2021).

### 2.2 Electrophysiological data source for AP recordings

Experimental intracellular recordings were obtained from two databases. The somatosensory cortex recordings came from the GigaScience database (da Silva Lantyer et al., 2018b), and the hippocampal neurons recordings came from the CRCNS database (Lee et al., 2016). The experimental procedures, such as temperature, electrode resistance, and perfusion conditions, for the somatosensory cortical recordings and data can be found in da Silva Lantyer et al. (2018a). The data were from current-clamp recordings of pyramidal regular-spiking (RS) neurons (*n* = 27) and fast-spiking (FS) neurons (*n* = 7). The experimental procedures for hippocampal neurons can be found in Lee et al. (2014, 2016). These current-clamp recordings from the17 RS pyramidal neurons were made from adult mice hippocampal CA1 neurons. The same AP selections criteria was used as previously described (Aldohbeyb et al., 2021), except the minimum inter-spike interval (ISI) was set to be 10 ms for all neuron types, which was used to separate APs during analysis. This limit did not exclude did not exclude many spikes across all neurons. Then, AP attributes were computed for responses to five current steps. First-AP attributes and average value was computed for each spike train. For each neuron type, we compared the AP attributes values elicited by the minimal current step that evoked a spike train against those elicited by each higher current step using a Mann–Whitney test. Significance level was adjusted for multiple comparison using the Bonferroni correction (α0.05/40.0125).

### 2.3 Computational models

All simulations presented here were done using MATLAB 2021, and the code can be found in GitHub (Combined Model). The dynamical equations were solved using the fourth order Runge-Kutta method with 1 μs time step, unless stated otherwise. The model describes a single-compartment conductance-based model for different cortical neuron types. The model includes Na^+^ and K^+^ voltage-gated channels and the membrane potential is described by the following equations (Pospischil et al., 2008):


$$C_{m}\,\frac{d\,V}{d\,t}=-g_{l\,e\,a\,k}\,\left(V-E_{l\,e\,a\,k}\right)+I_{N\,a}+I_{K\,d}+I_{a\,p\,p}$$

Where V is the membrane potential, *C*_*m*_ 1μF/cm^2^ is the specific membrane capacitance, *g*_*leak*_ and *E*_*leak*_ are the membrane conductance and its reversal potential calculated using Nernst equation at 34.1 degree Celsius. *I*_*Na*_ is the Na^+^ channels current, *I*_*Kd*_ is the “delayed-rectifier” K^+^ current. *I*_*app*_ is the applied (stimulus) current, which consists of 1 s step pulses separated by 6.5 s inter sweep interval, unless stated otherwise. The gating variables and their rate equations were used exactly as described in Pospischil et al. (2008) (Table 1).

**TABLE 1:** Channels’ rate equations.

Gating variables	α	β
*m*_*Na+*_	$\frac{0.32\,\left(V-V_{T}-13\right)}{1-\exp\left(-\frac{\left(V-V_{T-13}\right)}{4}\right)}$	$\frac{0.28\,\left(V-V_{T-40}\right)}{\exp\left(\frac{V-V_{T-40}}{5}\right)-1}$
*h*_*Na+*_	$0.128\,\exp\left(-\frac{V-V_{T}-17}{18}\right)$	$\frac{4}{\exp\left(-\frac{V-V_{T}-40}{18}\right)+1}$
*n*_*K+*_	$\frac{0.032\,\left(V-V_{T}-15\right)}{1-\exp\left(-\frac{V-V_{T}-15}{4}\right)}$	$0.5\,e\,x\,p\,\left(-\frac{V-V_{T}-10}{40}\right)$

#### 2.3.1 Cooperative sodium current

The voltage-dependent Na^+^ channel current was modified from a Hodgkin and Huxley (HH) type model as described by Pospischil et al. (2008) to include a fraction of cooperative Na^+^ channels. Cooperative Na^+^ channels followed Huang et al. (2012), where the activation of Na^+^ channels (*m*) was assumed to be instantaneous, and hence it was replaced by its steady-state value *m*. In this scheme, each channel is coupled to *K* neighboring channels, in which the opening of each of these channels shifts the channel’s activation curve toward more hyperpolarized potential by *J*mV. Therefore, the expected contribution of coupled channels scales with $K\,J\,m_{c}^{3}\,\left(V_{N\,a}\right)\,h$. When *J* 0*mV*, the model reduces to the classical case of independent Na^+^ channel activation, and when *J* 0*mV* Na^+^ channel activation becomes cooperative (Huang et al., 2012). The following equations describe the mean field approximation of the cooperative Na^+^ channels.


$$I_{N\,a}=\left(1-p\right)\,g_{N\,a}\,m_{\infty }^{3}\,\left(V\right)\,h\,\left(V-E_{N\,a}\,\left(t\right)\right)+p\,g_{N\,a}\,m_{c\,\infty }^{3}\,\left(V_{N\,a}\right)\,h\,\left(V-E_{N\,a}\,\left(t\right)\right)$$

$$m=\frac{\alpha_{m}}{\alpha_{m}+\beta_{m}}$$

$$\frac{d\,h}{d\,t}=\alpha_{h}\,\left(1-h\right)-\beta_{h}\,h$$

$$V_{N\,a}=V+K\,J\,m_{c\,\infty }^{3}\,\left(V_{N\,a}\right)\,h$$

Where *p* represents the fraction Na^+^ channels exhibiting cooperative gating and *KJ* is the coupling strength voltage between cooperative channels. α and β are the transition rate constants, which were adopted exactly as described in Pospischil et al. (2008). *E*_*Na*_ (*t*) represents the dynamic reversal potential for Na^+^.

Previous work reported that a small fraction of strongly coupled Na^+^ channels (*p* = 10–15%) with high coupling strength (*KJ* > 300*mV*) yielded the greatest AP rapidity and the largest threshold variability. Although this trend is consistent with experimental recordings, it was not reproduced in a single-compartment HH model. However, in our earlier analyses we found that the conclusion that a small fraction of cooperative channels maximizes rapidity arose from an artifact in the standard rapidity metric (see Aldohbeyb et al., 2020; Aldohbeyb, 2022), which was also discussed in Telenczuk et al. (2017). After correcting for this artifact, rapidity increases monotonically with both *KJ* and *p*, a result also confirmed using the IFWd^2^ rapidity measure. Accordingly, we used strong coupling (*KJ* > 400*mV*) and set half of the channels to be cooperative (*p* = 50%) to assess the maximal impact of cooperativity on spike train patterns, unless stated otherwise.

#### 2.3.2 Dynamical reversal potential

The model includes variable concentrations of intracellular and extracellular Na^+^ and K^+^, which are used to calculate the dynamic reversal potential (DRP) for each ion channel. The evolution of K^+^ concentration is determined, as described by Cressman et al. (2009), by the following equations:


$${\left[K^{+}\right]}_{i}=140\,m\,M+\left(18\,m\,M-{\left[N\,a^{+}\right]}_{i}\right)$$

$$\frac{d\,{\left[K^{+}\right]}_{o}}{d\,t}=\gamma \,B\,I_{K^{+}}-2\,B\,I_{p\,u\,m\,p}-I_{g\,l\,i\,a}-I_{d\,i\,f\,f}$$

$$I_{p\,u\,m\,p}=\left(\frac{\rho }{1+\exp\left(\left(25-{\left[N\,a^{+}\right]}_{i}\right)/3\right)}\right)\,\left(\frac{1}{1+\exp\left(5.5-{\left[K^{+}\right]}_{o}\right)}\right)$$

$$I_{g\,l\,i\,a}=\left(\frac{G_{g\,l\,i\,a}}{1+\exp\left(\left(18-{\left[K^{+}\right]}_{o}\right)/25\right)}\right)$$

$$I_{d\,i\,f\,f}=\varepsilon \,\left({\left[K^{+}\right]}_{o}-k_{o\,\infty }\right)$$

Here γ is a factor to convert current density to rate-of-change of concentration. *I*_*pump*_ represents the sodium-potassium pump. *I*_*glia*_ represents the capacity of glial cells to remove excess K^+^ from the extracellular space, and *I*_*diff*_ represents the diffusion of K^+^ away from the local extracellular micro-environment. The remaining factors and their values are described in Table 2. The equation for Na^+^ concentration was also adopted from Cressman et al. (2009). The Na^+^ concentrations are given by:


$$\frac{d\,{\left[N\,a^{+}\right]}_{i}}{d\,t}=\gamma \,I_{N\,a^{+}}-3\,I_{p\,u\,m\,p}$$

$${\left[N\,a^{+}\right]}_{o}=144\,m\,M-B\,\left({\left[N\,a^{+}\right]}_{i}-18\,m\,M\right)$$

**TABLE 2:** Model parameters and values.

Parameters	Value	Description
γ	0.33	Conversion factor from current density to rate-of-change of concentration (mM.cm^2^/μC)
*B*	7	Ratio of intracellular to extracellular volume of the cell
ρ	1.25	Pump strength (mM/s)
*G*_*glia*_	66.7	Strength of glial uptake (mM/s)
ε	1.2	Diffusion constant (s^–1^)
*k_o_*	4	Steady state extracellular potassium concentration (mM)
φ	0.35	Voltage dependence parameter
*E*_*Leak*_	-70	Leak channels reversal potential (mV)
*V_T_*	-63	Variable to adjust spike threshold (mV)
*g*_*Leak*_	0.15	Leak channels maximum conductance (mS/cm^2^)
*g*_*Na*_	50	Sodium channels maximum conductance (mS/cm^2^)
*g*_*Kd*_	10	Potassium channels maximum conductance (mS/cm^2^)
*L* = *W*	61.4	Length and width of the modeled membrane area (μm)

Finally, in some cases, we assumed that the available intracellular Na^+^ concentration might differ between the channels and Na/K pump. Thus, the concentration was adjusted to be:


$$\frac{d\,{\left[N\,a^{+}\right]}_{i}}{d\,t}=\gamma \,I_{N\,a^{+}}-I_{N\,a_{d\,i\,i\,f}}$$

$$\frac{d\,{\left[N\,a^{+}\right]}_{p\,u\,m\,p}}{d\,t}=I_{N\,a_{d\,i\,i\,f}}-3\,I_{p\,u\,m\,p}$$

$$I_{N\,a_{d\,i\,i\,f}}=\varepsilon_{N\,a}\,\left({\left[N\,a^{+}\right]}_{i}-{\left[N\,a^{+}\right]}_{p\,u\,m\,p}\right)$$

The intracellular Na^+^ concentration adjacent to the channels would increase while channel current flows to drive ion diffusion from the channel to the locations of Na/K pump proteins. Therefore, *I*_*Na_diif_*_ represents the diffusion current of sodium ions away from the channels to the nearest pump. The Na^+^ diffusion constant, ε_*Na*_, was obtained following Fick’s law (ε_*Na*_ = 2*D*_*Na*_/△*x*), where we used *D*_*Na*_ 0.3 μm^2^/ms and △*x* is the spacing between Na^+^ channels and the pump (Zylbertal et al., 2017). Here, the pump current is governed by the extracellular K^+^ concentration and Na^+^ concentration near the pump ([*Na*^+^]_*pump*_), instead of intracellular Na^+^ concentration near channels ([*Na*^+^]_*i*_). Note the Na^+^ concentration was assumed to be the same for the pump and channels in most sections of the results, unless stated otherwise.

## 3 Results

### 3.1 AP attributes evolve during continuous firing

**FIGURE 1:**
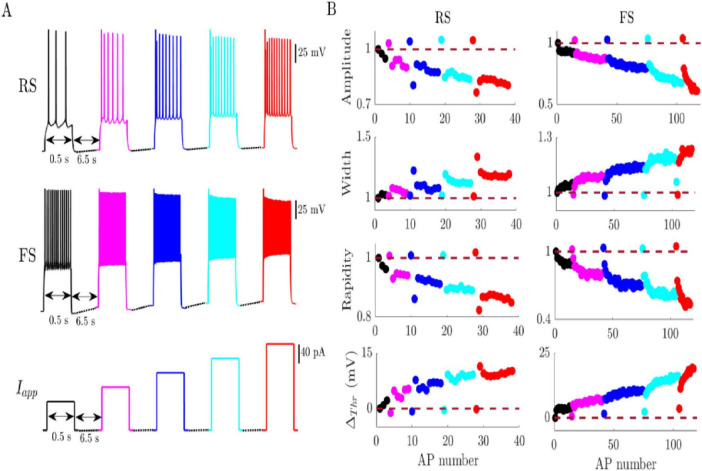
Changes in AP attributes of a typical regular spiking (RS) and a typical fast spiking (FS) somatosensory cortical neuron during whole-cell current-clamp recordings. **(A)** The spike trains evoked in typical RS and FS somatosensory cortical neurons by the current pulses (I_app_) shown at the bottom. The current stimulation protocol included 5 steps of 0.5 s long depolarization pulses increasing in magnitude (da Silva Lantyer et al., 2018a). **(B)** The attributes of each AP of the spike trains shown in A. The color of the spike train reflects the strength of the stimulus where black traces show the spike train evoked by the minimum current step and red shows the spike trains evoked by the highest current step. AP amplitude, width, and rapidity are normalized to the value of the first AP from the first pulse.

The shape of APs continuously changes during bursts evoked by injected current steps. The neuronal response is shown in terms of AP amplitude, width, rapidity, and threshold of recordings from three cell types: RS cortical neurons (*n* = 27), FS cortical neurons (*n* = 7), and RS hippocampal pyramidal neurons (*n* = 17). All the recordings analyzed show two distinct spike train features in response to current pulse stimulation. First, AP attributes monotonically change during spike trains evoked by each current pulse. Second, the first AP evoked by each current pulse has similar AP attributes independent of the stimulation strength. Figure 1 shows examples of RS and FS cortical neuron bursts in response to consecutive current step stimulations that increase in magnitude. The quantitative AP attributes, except threshold, are normalized to their first spike value evoked by the first current pulse. The threshold potential is shown as the threshold for each AP minus the threshold of the first AP in response to the first current pulse (△_thr_), so positive threshold indicates a more depolarized threshold and negative threshold change indicates a more hyperpolarized threshold compared to the first spike. A high current step depolarizes the membrane potential, leading to a higher average AP threshold potential for all APs within the burst, as expected. However, the threshold potential for the first AP remains almost unchanged, showing a minimal increase compared to the threshold potentials of the subsequent APs in the burst. For example, the largest change in the threshold of the first APs of each step is only 4 mV, while the average threshold for the fifth spike train was 15 mV higher than the first spike train. Likewise, the first spike evoked by the fifth (red) current step in cortical FS neuron was only 4.5% more rapid than the first spike evoked by the first (minimum) current step. In contrast, the rapidity of the last spike in the fifth current step was 45% slower in rapidity compared to the first spike of the first current pulse. Spike train patterns similar to those shown in Figure 1 were observed in all 51 neuron recordings analyzed here.

The presence of the two distinctive spike train patterns, one for the first spike and another for subsequent spikes, across multiple neuron types suggest a shared mechanism responsible for such neuronal behavior. Although the absolute magnitude of neuronal response before normalization varies between individual neurons, the normalized patterns are similar for all analyzed neurons (*n* = 51). The first spikes’ attributes are clustered around the same value, similar to that of the initial stimulus pulse, despite the increasing stimulus strength. This makes the magnitude variability between individual neurons more distinct when considering the average or final values of the AP bursts. For instance, in RS cortical neurons, the rapidity of the first AP in the first and fifth spike trains is statistically insignificant (Mann Whitney *z* = 0.77, *p* = 0.4, common language effect size, CLES = 0.44). However, the average rapidity of the fifth spike train is significantly lower than that of the first spike train (Mann Whitney *z* = 3.06, *p* = 0.002, CLES = 0.74). Similar AP attribute patterns were observed for FS cortical neurons and RS hippocampal neurons. While the statistical comparison in FS cortical neurons showed no significant differences in the average for most AP parameters, the observed trends still indicate a monotonic change in average AP attributes with stimulus strength. The average AP amplitude and rapidity decrease, and the average AP threshold and width increase during the multiple step stimulation protocol. The average intra-pulse AP attribute variation also increases with each subsequent spike train. Tables 3–5 summarizes the mean and standard deviation values for each of the three neuron types in response to five current steps. Although the examined recordings consistently exhibit these AP attribute patterns, classical models fail to reproduce them, as will be discussed next.

**TABLE 3:** AP attributes of FS cortical neurons (*n* = 7) in response to five current steps (mean ± SD).

I_app_ Step	Rapidity (ms^–1^)		Threshold (mV)		Amplitude (mV)		Width (ms)	
	1st APs	Average	1st APs	Average	1st APs	Average	1st APs	Average
1st	3.10 ± 0.3	2.97 ± 0.3	–39.2 ± 8.1	–35.2 ± 7.0	63.0 ± 4.9	57.5 ± 4.2	0.66 ± 0.09	0.66 ± 0.10
2nd	3.14 ± 0.3	2.87 ± 0.4	–40.7 ± 8.1	–34.2 ± 7.4	66.4 ± 1.2	56.4 ± 3.6	0.65 ± 0.08	0.67 ± 0.10
3rd	3.17 ± 0.3	2.77 ± 0.4	–40.2 ± 8.3	–32.6 ± 8.0	66.7 ± 1.1	54.1 ± 3.5	0.65 ± 0.08	0.69 ± 0.11
4th	3.17 ± 0.3	2.67 ± 0.5	–39.9 ± 8.7	–30.5 ± 8.9	67.1 ± 1.3	51.3 ± 4.1	0.65 ± 0.08	0.70 ± 0.12
5th	3.19 ± 0.2	2.56 ± 0.5	–39.2 ± 8.9	–28.3 ± 10	67.3 ± 1.2	48.2 ± 5.3‡	0.65 ± 0.08	0.72 ± 0.12

‡ indicate significant different using Mann Whitney z at α = 0.0125.

**TABLE 4:** AP attributes of RS cortical neurons (*n* = 27) in response to five current steps (mean ± SD).

I_app_ Step	Rapidity (ms^–1^)		Threshold (mV)		Amplitude (mV)		Width (ms)	
	1st APs	Average	1st APs	Average	1st APs	Average	1st APs	Average
1st	2.84 ± 0.3	2.57 ± 0.3	–34.5 ± 9.2	–28.9 ± 8.8	79.6 ± 7.8	69.3 ± 9.2	1.43 ± 0.25	1.54 ± 0.26
2nd	2.86 ± 0.2	2.47 ± 0.3	–34.1 ± 9.90	–26.2 ± 9.3	80.7 ± 7.7	65.7 ± 8.3	1.42 ± 0.26	1.62 ± 0.29
3rd	2.89 ± 0.2	2.37 ± 0.3	–34.1 ± 10.1	–23.8 ± 9.5‡	81.6 ± 8.1	61.4 ± 9.1‡	1.42 ± 0.26	1.68 ± 0.29
4th	2.90 ± 0.2	2.32 ± 0.4‡	–33.2 ± 10.7	–22.1 ± 10.9‡	81.9 ± 8.3	59.5 ± 10.7‡	1.42 ± 0.26	1.72 ± 0.26‡
5th	2.90 ± 0.2	2.26 ± 0.3‡	–32.4 ± 11.1	–20.1 ± 11.5‡	81.7 ± 8.6	56.2 ± 11.4‡	1.42 ± 0.26	1.75 ± 0.26‡

‡ indicate significant different using Mann Whitney z at α = 0.0125.

**TABLE 5:** AP attributes of RS hippocampal neurons (*n* = 17) in response to five current steps (mean ± SD).

I_app_ Step	Rapidity (ms^–1^)		Threshold (mV)		Amplitude (mV)		Width (ms)	
	1st APs	Average	1st APs	Average	1st APs	Average	1st APs	Average
1st	5.53 ± 0.6	5.20 ± 0.5	–36.5 ± 2.7	–35.6 ± 2.7	78.5 ± 7.4	76.6 ± 6.8	1.04 ± 0.08	1.07 ± 0.08
2nd	5.54 ± 0.5	4.84 ± 0.6	–35.5 ± 2.8	–33.6 ± 2.8	77.1 ± 7.4	73.2 6 ± 6.6	1.03 ± 0.07	1.15 ± 0.10‡
3rd	5.57 ± 0.4	4.64 ± 0.7	–35.1 ± 2.8	–32.1 ± 3.0‡	75.8 ± 7.6	71.0 6 ± 6.5	1.02 ± 0.08	1.26 ± 0.15‡
4th	5.45 ± 0.5	4.51 ± 0.7‡	–34.5 ± 3.1	–30.8 ± 3.4‡	74.9 ± 7.6	69.26 ± 6.5‡	1.00 ± 0.08	1.38 ± 0.20‡
5th	5.51 ± 0.6	4.38 ± 0.8‡	–34.0 ± 3.4	–29.4 ± 3.7‡	74.2 ± 7.7	67.56 ± 6.6‡	1.00 ± 0.08	1.50 ± 0.25‡

‡ indicate significant different using Mann Whitney z at α = 0.0125.

### 3.2 Ion concentration changes dictate monotonic variation in AP attributes

**FIGURE 2:**
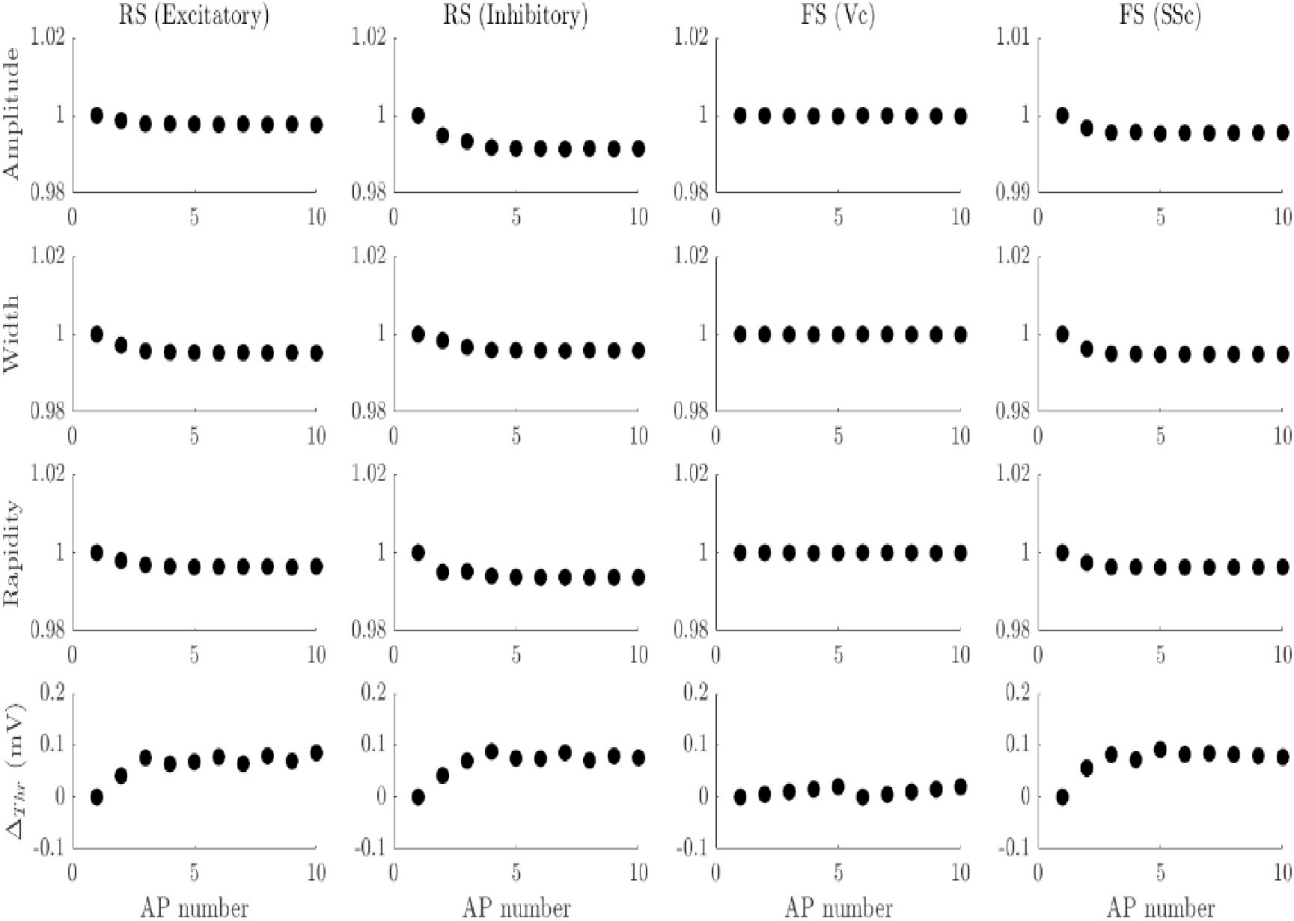
AP attribute evolution in spike trains in different HH-type models. All the models are used as described in Pospischil et al. (2008). The models include three ionic currents (*I*_*Na^+^*_, *I*_*K^+^*_, and *I_M_*), except the FS model for a visual cortical neuron (Vc) that includes only *I*_*Na^+^*_ and *I*_*K^+^*_. The variation of normalized AP amplitude, width, and rapidity is less than 1% for all models.

A variety of HH models for different classes of neurons produce different AP shapes and firing patterns but fail to replicate the variation in AP attributes during bursts. Simple models of FS cortical neurons [incorporating only sodium current (I_*Na*^+^_), and delayed-rectifier potassium current (I_*K*^+^_] were sufficient to generate the intrinsic firing characteristics of experimental recordings (Pospischil et al., 2008). In some cases, it was necessary to add slow non-inactivating potassium current (I_*M*_) channels for initial frequency adaption observed in several neuron types (Pospischil et al., 2008). Furthermore, Additional models to mimic RS excitatory and inhibitory cortical neurons, intrinsically bursting (IB) neurons, which included more ion channels (Pospischil et al., 2008). However, all these models fail to replicate the monotonic variation in AP attributes observed in intracellular recordings (Figure 2; Supplementary Figure 1). Although the threshold onset potential increases within the first few APs due to I_*M*_, the threshold stays constant for the remaining APs within a burst. Various HH model types with different rate functions were investigated as well, but none of these models reproduce the AP attributes observed in trains of APs, indicating that key mechanisms responsible for explaining such variation are missing in the classical models.

**FIGURE 3:**
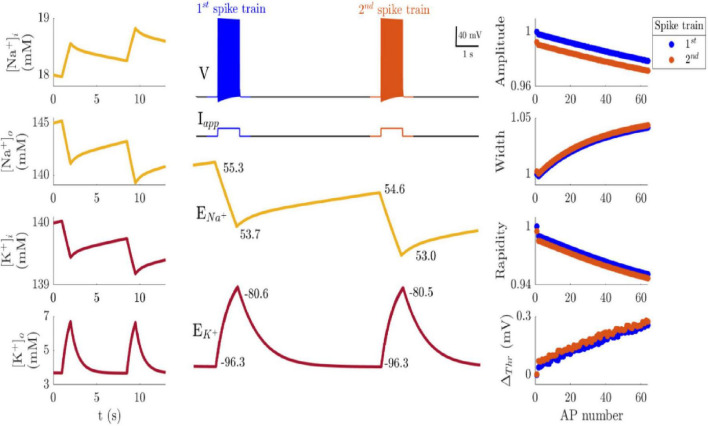
Membrane potential (V), and reversal potentials (*E*_*Na+*_ and *E*_*K+*_) change during two current pulses (middle, *I*_*app*_ = 3.5 μA/cm^2^). The left column shows the changes in Na^+^ and K^+^ concentration inside and outside the cell. The right column shows four attributes of each AP as well as the firing rate during the first (blue) and second (red) current pulse. Note that the change in *E*_*Na+*_ during the stimulus pulse does not recover to within 1% of its resting value during the 6.5 s between pulses, unlike the change in $E_{K^{+}}.$

Sustained variation in AP attributes emerges when dynamic reversal potentials (DRPs) are incorporated into any of the HH models described above. Therefore, we used FS cortical neuron model throughout the paper to demonstrate the effects of DRP, cooperativity, or both on spike train patterns. Figure 3 shows spike trains evoked by two identical current pulses separated by a 6.5-s interval, similar to the timing used in intracellular recordings of RS and FS cortical neurons in da Silva Lantyer et al. (2018a).

The absolute change in ion concentration was almost identical in the two bursts since the same stimulus is applied. For example, intracellular Na^+^ concentration ([Na^+^]_i_) and extracellular K^+^ concentration ([K^+^]_o_) increased by the same amount during the two spike trains [0.6 mM for [Na^+^]_i_ and 3 mM for [K^+^]_o_]. However, although the change in [Na^+^]_i_ is small, its removal is slow, while the change in [K^+^] is large but rebalances faster after excitation. As a result, a rise in ${\text{E}}_{{\text{K}}^{+}}$ by 16 mV at the end of the current pulses decays back to near the steady-state value within 3 s since (∼1 s time constant). At the same time, ${\text{E}}_{{\text{Na}}^{+}}$ drops by a much smaller value (∼ 1.6 mV), but slowly increases and only recovered by 48% after 6.5 s (∼ 9 s time constant). Consequently, the change in ${\text{E}}_{{\text{K}}^{+}}$ and ${\text{E}}_{{\text{Na}}^{+}}$ alter the AP shape features differently, as shown in Figure 3. The change in AP width was almost identical in the two bursts (4.3% increase) since it is mainly controlled by K^+^ channels. On the other hand, the Na^+^ current contributes to the difference in the changes of the other AP attributes between the two bursts. Thus, there is a slight shift in the AP amplitude, rapidity, and threshold in the second spike train (Figure 3, red symbols). Therefore, the systematic variation in the AP attributes is due to ${\text{E}}_{{\text{Na}}^{+}}$ and ${\text{E}}_{{\text{K}}^{+}}$ change during the pulse, and the slow recovery of ${\text{E}}_{{\text{Na}}^{+}}$leads to the first AP attributes’ reduction of the subsequent burst. The slight reduction of the first APs of subsequent spike trains diverges from experimental data where AP initiation attributes slightly increase (Tables 3–5). However, inclusion of DRP causes modeled AP attributes to vary in a manner resembling experimental data during a step-current protocol.

### 3.3 Cooperativity and DPR replicates the spike patterns observed in intracellular recordings

**FIGURE 4:**
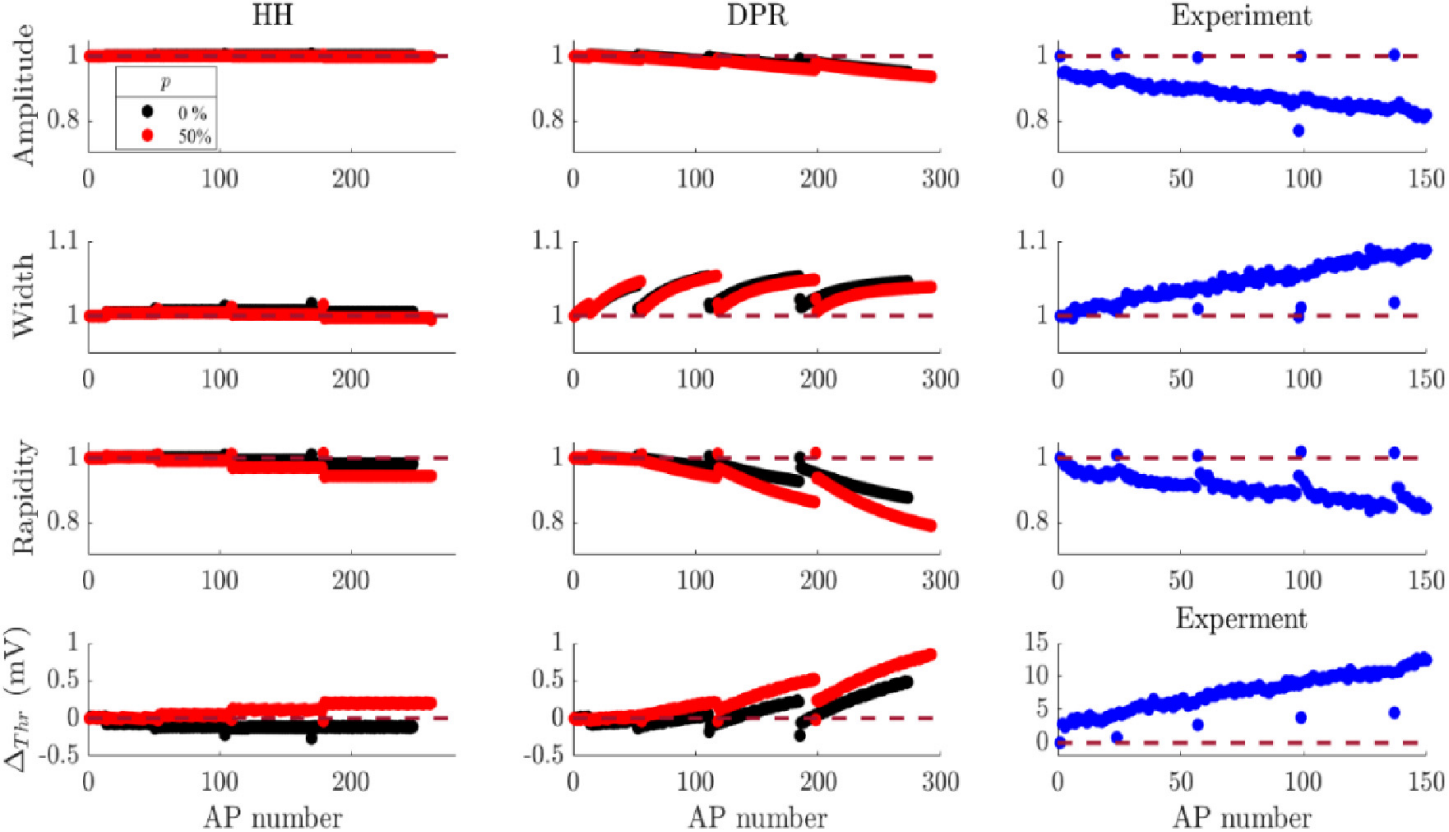
Comparison between AP attributes from different models and experimental recordings. Left: the model was run with fixed reversal potentials; black circles represent the values obtained from the original HH model, and red circles represent the values obtained from the cooperative channels model (*p* = 50% and KJ = 400 mV). Middle: the model with dynamical reversal potential with independent Na^+^ channels (black circles), and with 50% cooperative Na^+^ channels (red circles, KJ = 400 mV). The modeled stimulation was five 1 s-current steps (1.6, 2.4, 3.2, 4, and 4.8 μA/cm^2^) with 6.5 s inter sweep interval. Right: AP rapidity and threshold difference from a somatosensory FS cortical neuron in response to five current steps (0.5 s steps of 80, 120, 160, 200, and 240 pA with 6.5 s intra sweep interval) (da Silva Lantyer et al., 2018b).

Cooperative Na^+^ channel gating enhances the variation of AP initiation attributes (threshold voltage and rapidity) during spike trains. Inclusion of DRP alone causes monotonic variation in AP attributes, but the magnitude of modeled variation is smaller than the experimental data, as shown above. Combining Na^+^ channel cooperativity and DRP increases the magnitude of modeled variation in all AP attributes except AP width. Although the magnitude of modeled threshold change is still small compared to experimental data, the magnitude of changes in AP initiation attributes almost doubled in the presence of strong cooperative Na^+^ gating (Figure 4 and Supplementary Figure 2). Cooperativity boosted DPR change so that APs are fired at a less depolarized (higher) threshold voltage and with reduced rapidity in response to a strong stimulus. For example, with cooperativity the last AP (I_app_ = 4.8 μA/cm^2^) was triggered at a 0.85 mV higher threshold and with 21% lower rapidity than the first AP (I_app_ = 1.6 μA/cm^2^), compared to 0.49 mV higher threshold and 12% rapidity reduction without cooperativity. The impact of cooperative Na^+^ channels on the first spike attributes diverges from the classical HH model. Cooperative Na^+^ channel gating kept the first APs shape comparable despite the strength of the stimulus. Figure 4 illustrates the difference between the models’ impact on AP features, compared to experimental recordings. First APs are initiated at a more hyperpolarized membrane potential in response to strong stimulus in the HH model. The relationship between stimulus strength and threshold is reversed in the presence of cooperative Na^+^ channels where first spikes are triggered at the same threshold voltage. Likewise, the first APs’ rapidity slightly increases with each current step with cooperative gating, resembling experimental data. The first AP of the fifth spike train has 1.5% higher rapidity than the first AP from of first spike train (red Figure 4), which is similar to the FS recordings (1.6% increase in Figure 4, blue). The increase in the modeled rapidity of first APs is almost the same with or without DRP. However, the systematic attribute variation disappears without DRP. Therefore, the results show that both observed spike train features are only replicated by combining DRP and cooperative Na^+^ channel gating, where the magnitude of modeled variation agrees with experimental observations for some AP attributes. However, even with the variation increase due to cooperativity, the model still underestimates the threshold variation observed in intracellular recordings by more than a factor of 10.

### 3.4 Cell volume and pump strength effect on AP attributes

The magnitude of AP attributes variation differs significantly between individual neurons. For example, the reduction in average rapidity ranges from 10 to 43% in the recordings from FS cortical neurons. The spread in the magnitude of monotonic variation was also observed in hippocampal and cortical RS neurons, indicating that cell-dependent factors contribute to the observed attribute variation. Neuron volume changes during abnormal and normal activities, and even neuron dilation during an individual AP has been estimated (Iwasa et al., 1980; Ullah et al., 2015), where the neuron size stability is regulated by ion pumps and intracellular and extracellular osmolarity (Kay, 2017). Thus, the volume ratio of intracellular to the extracellular, Na/K pump strength, and Na^+^ concentration near the pump were adjusted to explore their impact on spike train patterns.

Altering cell volume ratio significantly impacts the magnitude of modeled AP attributes variation. The rate of ion concentration change is directly impacted by the intracellular volume to the extracellular volume ratio. Increasing the volume ratio did not influence the first AP of each pulse but did lead to higher variation of AP attributes during pulses (Supplementary Figure 3). The impact of volume ratio also increases with cooperativity. For instance, doubling the volume ratio from 7 to 14 causes the rapidity to drop from 11 to 22% without cooperative channels and from 20 to 37% with half the Na^+^ channels activated cooperatively. However, such a notable difference existed only with unrealistic volume changes such as a 50% increase in the cell volume ratio.

The Na/K pump strength was modeled with two extreme values in addition to the nominal value to explore the Na/K pump’s impact on AP onset dynamics. An order of magnitude increases in Na/K pump speed made minimal changes to predicted AP initiation attributes while an order of magnitude decrease led to either much smaller variation of attributes—and thus widened disagreement with experimental observations—or in the absence of cooperativity led to pathological behavior of runaway ion concentration changes (Supplementary Figure 4).

**FIGURE 5:**
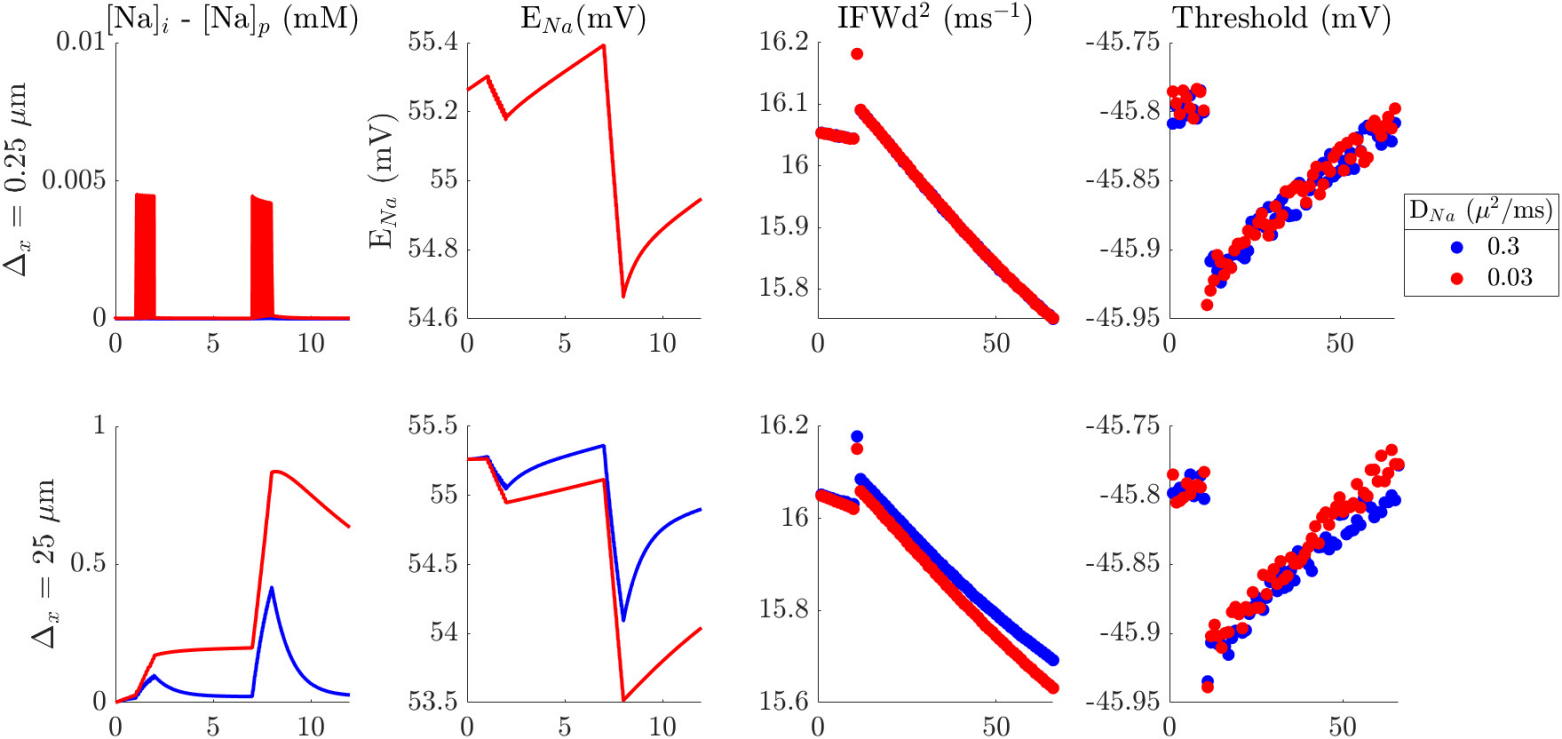
Impact of spacing difference between the pump and Na^+^ channels and diffusion coefficient on concentration with no cooperative gating. Na^+^ concentration difference between the pump and Na^+^ channels (first column), its effect on Na^+^ channel reversal potential (second column), AP rapidity (third column), and AP threshold potential (fourth column). Blue traces and circles indicate the value when using the reported diffusion coefficient (*D*_*Na*_ = 0.3μm^2^/ms). red traces and circles indicate the value when using slow diffusion coefficient (*D*_*Na*_ = 0.03μm^2^/ms). The spacing between the channels and the pump was 0.25 μm (top row), or 25 μm (bottom row). Supplementary Figure 6 shows the effect with cooperative gating, which also did not lead to any significant difference in AP initiation attributes.

Furthermore, the impact of Na^+^ diffusion between channels and the pump was investigated to see if it could account for the underestimation of changes in threshold potential and AP amplitude. The model was modified to track two different intracellular Na^+^ concentrations: [Na^+^]_i_ at Na^+^ channels and [Na^+^]_pump_ at Na/K pumps. However, even across a broad range of slow Na^+^ diffusion constants and larger spacing between the channels and the pumps, the difference in Na^+^ concentration between the two types of sites was too small to cause any noticeable effect on AP attributes (Figure 5 and Supplementary Figures 5, 6). Therefore, even if there might be a difference in local Na^+^ concentration between intracellular domains near to pump vs. channels, our simulations did not reveal any resulting change in AP attributes.

## 4 Discussion

Here, we observed two key spike train patterns from electrophysiological recordings of cortical and hippocampal neurons: AP shape attributes show monotonic changes during step-current pulses, while the first spikes of each train remain consistent across different stimulus intensities. To explain these patterns, we developed a single-compartment conductance-based model that incorporates the dynamics of ion concentration and cooperative Na^+^ channel gating. Our analysis shows that the combined model reproduces the spike train pattern observed in central mammalian neurons, where we examined the impact of several factors on neuronal dynamics. The results show that the dynamic change of reversal potential due to changes in the local ion concentration alters the AP attributes during repetitive spike trains, and the level of change, for some AP attribute, is enhanced in the presence of a fraction of cooperative Na^+^ channels.

Understanding the mechanisms of AP initiation is an integral part of determining how neurons encode information. The variability in neuronal response was observed between individual neurons of the same cell type (Goldman et al., 2001), and for the same neuron in response to the same DC stimulus (Mainen and Sejnowski, 1995). The observed variability of neuronal response could arise from several factors such as the diversity in voltage-gated ion channel types and densities, diversity of synaptic inputs, or the neuron intrinsic properties (Mainen and Sejnowski, 1995; Marder and Goaillard, 2006). While repetitive DC current stimulation has been shown to produce imprecise spike timing across trials, examining AP shape attributes under the same conditions reveals consistent and systematic patterns, but in-term of AP shape features not spike timing. These two observations may reflect the same underlying biophysical processes, viewed from different angles—one temporal, the other morphological. For example, in 27 regular-spiking (RS) cortical neurons, the rapidity of the first APs was tightly clustered (2.90 ± 0.2 ms^–1^) and approximately 28% higher than the average AP rapidity (2.26 ± 0.3 ms^–1^) observed under strong stimulation (Table 4). These findings align with observations by Mainen and Sejnowski, who reported precise timing of initial spikes and greater variability in later ones (Mainen and Sejnowski, 1995). While their focus was on spike timing, we found a similar trend in terms of AP shape: repeated constant DC stimulation produced reproducible first AP shapes but increasingly variable later spikes, while keeping the same pattern (Figure 1 and Supplementary Figure 7). As shown in Supplementary Figure 7, repeating the same DC stimulation protocol resulted in consistent AP shape patterns, with slight shifts across trials. These small drifts in AP attributes suggest that the same biophysical processes contributing to spike timing variability could also underlie the observed variability in AP waveform. However, the datasets used in this study did not include sufficient repetition of identical stimuli across all neurons to fully confirm this connection. Nonetheless, this observation highlights an interesting avenue for investigating neural variability in terms of both spike timing and AP shape dynamics.

To reproduce the experimentally observed spike train patterns, we incorporated dynamic ion concentration changes into the computational model. Neither the classical HH model nor its cooperative Na^+^ channel variant could replicate these patterns without including DRPs. The rationale for using DRPs is that ionic currents partially deplete or accumulate ion concentrations in the vicinity of ion channels when those concentrations are restored via diffusion rather than directly fixed to bulk reservoir concentrations. The shift in ion concentrations adjacent to channels during current pulses can alter the channels’ reversal potential, which in turn affects neuronal excitability. Clusters of voltage-gated ion channels, which exist in various neuronal regions, can further amplify local ion concentration changes. Such amplification is particularly strong when multiple channels within a cluster open simultaneously. In the case of cooperative Na^+^ channels, such synchronized activity has been proposed to underlie the rapid AP initiation seen in cortical neurons (Naundorf et al., 2006; Huang et al., 2012). This suggests a potential interplay between ion concentration dynamics and cooperative channel gating. The interdependence of ion concentration dynamics on more rapid changes in membrane voltage associated with cooperative channel gating, and the evolution of cooperative gating in the presence of altered ion concentrations, raise an interesting question about how the combination of cooperativity and ion concentration changes modifies neuronal firing patterns. The impact of concentration change depends primarily on the instantaneous magnitude of ionic current (as described in the Methods), which is enhanced by cooperative gating. Thus, the combined effect of Na^+^ channel cooperativity and DRP is analyzed to investigate how the two mechanisms alter AP attributes and firing patterns compared to classical HH and cooperative models, and how these changes fit the observed experimental data.

The proposed model reproduced the overall patterns observed in experimental data, although the magnitude of change varied across AP shape attributes, with some features more accurately captured than others. The shape of APs differs significantly between neuron types. FS neurons exhibit narrower AP width and higher frequency than RS neurons (McCormick et al., 1985; Nowak et al., 2003; Carter and Bean, 2009). Prior studies associated the activity-dependent decrease in AP amplitude and broadening AP width to Na^+^ and K^+^ channels (Gillette et al., 1980; Jackson et al., 1991; Geiger and Jonas, 2000; De Polavieja et al., 2005), where each type of channel influences the spike waveform differently. The slow K^+^ channel inactivation recovery was shown to be the primary factor in AP broadening (Geiger and Jonas, 2000), similar to the observed AP width pattern shown above (Figure 1). While our model underestimated the observed percentage of AP broadening, the results indicate that changes in K^+^ concentration could be among the factors contributing to AP broadening during firing. Furthermore, the insensitivity of AP width to Na^+^ channel cooperativity is thus expected since cooperativity is expected to increase ${\text{I}}_{{\text{Na}}^{+}}$, whereas the AP width is mainly determined by ${\text{I}}_{{\text{K}}^{+}}$ (Lien and Jonas, 2003; Bean, 2007). In contrast, reduction in AP amplitude was associated to blocking voltage-dependent calcium current in Purkinje cells (Raman et al., 1997), and by Na^+^ channel activation in cortical pyramidal neurons (Stuart and Sakmann, 1995). A previous study using multicompartment models that included only Na^+^ and Ca^2+^ concentration dynamics was able to reproduce amplitude adaptation in a cortical pyramidal cell model, but not in a Purkinje cell model (Zylbertal et al., 2017). Here, we used a simple cortical neuron model that includes Na^+^ and K^+^ concentration dynamics to reproduce similar AP broadening and amplitude reduction. Although the magnitude of modeled variation is smaller than the observed experimental change, ion concentration dynamics could be another factor effecting AP width and amplitude patterns, but not the only factor based on the modeling scheme using in this study.

The impact of DRP is more significant on AP initiation attributes, especially rapidity, than AP amplitude and width. Previous studies had linked the threshold variation to the rate of membrane potential changes prior to the AP, stimulus history, Na^+^ channel density and activation or inactivation processes, or AP backpropagation (Azouz and Gray, 2000; Henze and Buzsáki, 2001; De Polavieja et al., 2005; Kole et al., 2008). The mechanism behind high variation in AP initiation attributes was described to must have two criteria: activated by preceding individual APs and have long recovery time, which are characteristics of Na^+^ channels kinetics (Henze and Buzsáki, 2001). These criteria also apply to the dynamics of ion concentration, especially Na^+^, that are changed by individual APs and have slow recovery (Figure 3). Moreover, higher threshold was found to coincide with slower spike rise (the slope of the AP from resting potential to the AP peak) as well, which is an indication of the degree Na^+^ channel inactivation (De Polavieja et al., 2005). Our analysis of intracellular recordings agrees with these observations that higher threshold coincides with lower AP rapidity. A simple one-compartment model that includes DRP replicates the relationship between threshold and rapidity and shows a good correspondence with experimental observations for AP rapidity, but not the size of threshold change. Specifically, the modeled threshold variation was approximately tenfold smaller than that measured experimentally. This discrepancy suggests that additional mechanisms not included in the proposed model such as AP backpropagation from the axon initial segment (AIS) (Yu et al., 2008), Na^+^ channel inactivation kinetics (Henze and Buzsáki, 2001), coupling resistivity between the soma and AIS (Brette, 2013), may contribute to the observed threshold changes. On the other hand, the magnitude of simulated rapidity variation was within the lower range of the observed change in experimental recordings, indicating that ion concentration changes could be responsible for the variation in AP initiation, and hence influencing neuronal excitability during normal neuronal dynamics.

Combining cooperativity and dynamical reversal potential enhances the magnitude of modeled variation and offsets the slight decrease in the first spikes rapidity. Cooperative Na^+^ channels were proposed to explain the highly variable AP onset and sharp rapidity in cortical neurons (Naundorf et al., 2006). Thus, cooperative gating can set the level of AP initiation attributes, and their impact on ion concentration dynamics increases the magnitude of variation during bursts. The two bursts’ features could not be replicated in a variety of HH-type models without including DRP and cooperative gating. Cooperativity has a more substantial impact on some of the AP attributes, but not all. The variation in AP rapidity, in particular, increases with cooperative gating to fall within the range of experiential observation. For example, making 50% of sodium channel activate cooperatively, with DRP, approximately doubled the reduction in modeled rapidity compared to the case of no cooperativity. Thus, combining cooperativity and DRP increased the magnitude of simulated rapidity variation to be around the average change in experimental recordings (Tables 3–5).

Finally, investigating the impact of the Na/K pump strength, distance from Na^+^ channels, and the cell volume ratio did not substantially improve our model. Altering the pump strength led to a small change in the spike train pattern in the case of a slow pump or disrupting the pattern in the case of a fast pump, thus, widening disagreement with experimental observations. Moreover, the spacing between the sodium channels and the Na/K pump, as well as the diffusion coefficient, had an insignificant effect on sodium concentration dynamics, regardless of the presence of cooperative gating channels. Increasing the distance between channels and pumps produced no noticeable changes in spike-train patterns or their average values in the current model. These findings suggest that local concentration changes arising from the distance between channels and pumps are minimal and therefore exert negligible influence on the reversal potential and, subsequently, on the rapidity and threshold of neuronal firing. This may imply that clustering channels near pumps, which accelerating the replenishment of the local sodium near the channels, does not substantially affect AP initiation parameters and that an additional mechanism is missing from the present model. Alternatively, it may indicate that the simplified framework used here underestimates the role of ion concentration dynamics, and that a more detailed formulation, such as a Poisson–Nernst–Planck model, is required to fully capture their impact AP properties and spike-train patterns.

On the other hand, increasing the volume ratio enhanced the magnitude of variation, which is expected since the volume ratio directly affects the ion concentration dynamics (see Methods). However, for the normal firing behavior presented here, an unrealistically large change in cell volume (such as increasing the volume ratio by 50%) is needed to observe any substantial difference in the neuron dynamics. A 60% swelling was observed in pyramidal cortical neurons during anoxic depolarization (Devin Brisson and David Andrew, 2012), and even a 10% change in the cell diameter can lead to seizure-like events (Ullah et al., 2015). Furthermore, pyramidal neurons were shown to maintain their volume during normal condition (Andrew et al., 2007). However, other studies have used larger volume ratio than the value used in our proposed model here (β = 7 following Cressman et al., 2009) such as 10 in Chizhov et al. (2018) and 20 in Chizhov and Sanin (2020). These larger values increased the magnitude of AP variation in, but threshold variation remained small compared with experimental data. Therefore, from a modeling perspective, changes in the volume ratio can influence normal neuronal firing behavior and its effect increases in the presence of cooperative channels. These changes, however, underestimates threshold variation. This suggests that while DRP replicates spike train patterns, the underlying mechanism for threshold variation is missing.

## 5 Limitation

This study has several limitations. First, recordings from the different neuron types were obtained from open-source databases, so the sample size for each neuron type could not be increased. The sample size for RS cortical (*n* = 27) and hippocampal neurons (*n* = 17) was larger than for FS cortical neurons (*n* = 7). Therefore, although spike-train patterns are shown for FS neurons, differences in subgroup comparisons (between step currents) should be interpreted cautiously, as true effects may be missed and the results may not generalize reliably for FS neurons. Second, we used a simple one-compartment model that did not account for realistic neuronal morphology, such as AP initiation in the axon initial segment (AIS), or the size differences between the soma, axon, and dendritic tree. These morphological factors were previously shown to impact AP shape parameters (Yu et al., 2008; Eyal et al., 2014; Telenczuk et al., 2017). Third, although we utilized HH models with various voltage-gated channels (as described in Pospischil et al., 2008), other channels like calcium-dependent K+ channels or HCN channels were not included and might alter the observed spike train pattern. Finally, the model understates the complexity of ion concentration dynamics. While Electrodiffusion or Kirchhoff-Nernst-Planck models could offer a better description, they are computationally expensive compared to the scheme used here (Solbrå et al., 2018; Savtchenko et al., 2017). Finally, although our proposed model produced patterns similar to experimental results, it lacked experimental confirmation of its underlying assumptions. Future studies that experimentally modify sodium and potassium concentrations during a current clamp protocol are necessary to validate the influence of ion concentration on the observed spike train pattern. Thus, further studies are required to fully understand the effects of ion concentration on the spike train observed during the step-and-hold protocol.

## 6 Conclusion

Analysis of publicly available experimental recordings for three different neuron types show that AP attributes monotonically change during bursts while the first APs of each burst have comparable AP attributes. A single compartment model that includes DRP replicated some of the observed trends in AP attributes, and adding cooperative Na^+^ gating enhanced those trends. The magnitude of modeled variation agrees with experimental observations for some AP attributes (rapidity, width) but not threshold and amplitude. Threshold variation, in particular, was underestimated by the model, indicating that the current model is missing the mechanism that captures the observed trend. Further investigations are needed to resolve this disagreement.

## Data Availability

The original contributions presented in the study are included in the article/Supplementary material, further inquiries can be directed to the corresponding authors.
