# Supplementary material for: The impact of dynamic reversal potential on the evolution of action potential attributes during spike trains
Source: Front Comput Neurosci. 2026 Jan 9;19:1740570. doi: 10.3389/fncom.2025.1740570 (PMC12827510; doi:10.3389/fncom.2025.1740570)
Supplement: Supplementary file 1 [file Table_1.docx]

# **Supporting information**


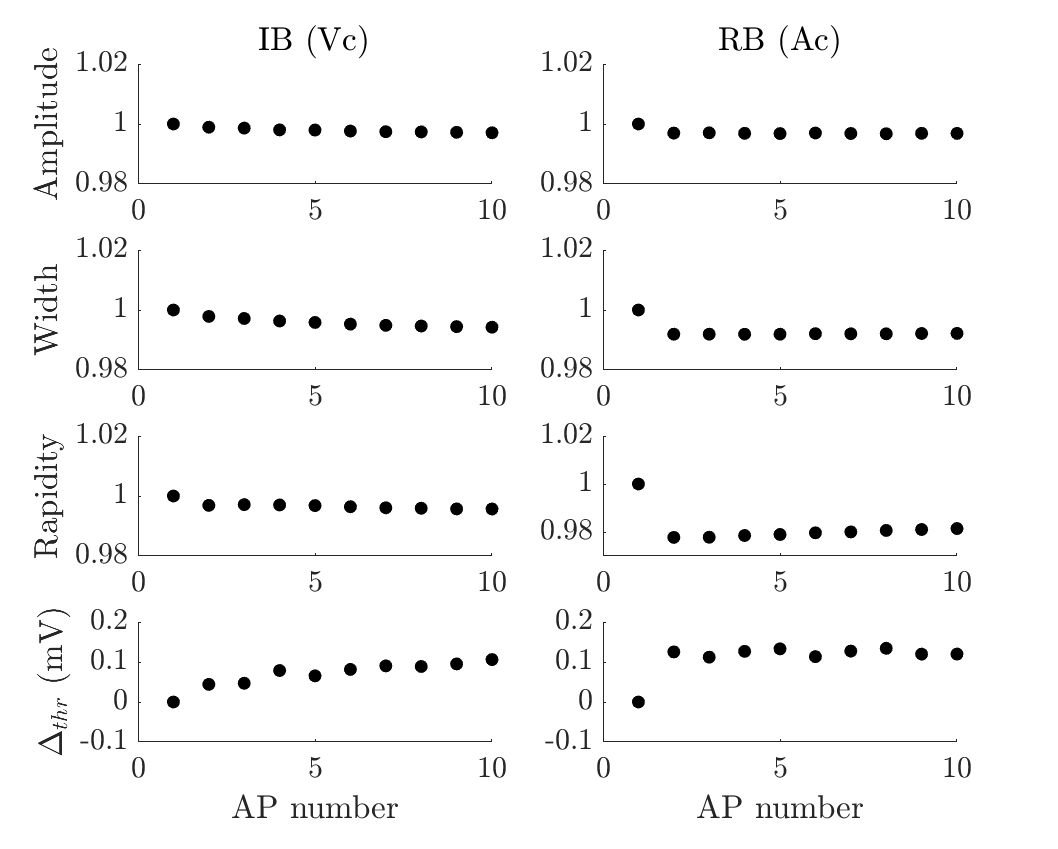


**Figure S1:** **AP attribute evolution in spike trains in different HH-type models.** All the models are used as described in Pospischil et al. (2008). Left: the model for intrinsically bursting (IB) based on cat visual cortex (Vc). Right: the model for rebound bursting (RB) neuron based on cat association cortex (Ac). The models include different ionic currents ( $I_{{Na}^{+}}$, $I_{K^{+}}$,$I_{M}$, $I_{Ca_{H}^{+}}$ , or $I_{Ca_{L}^{+}}$).


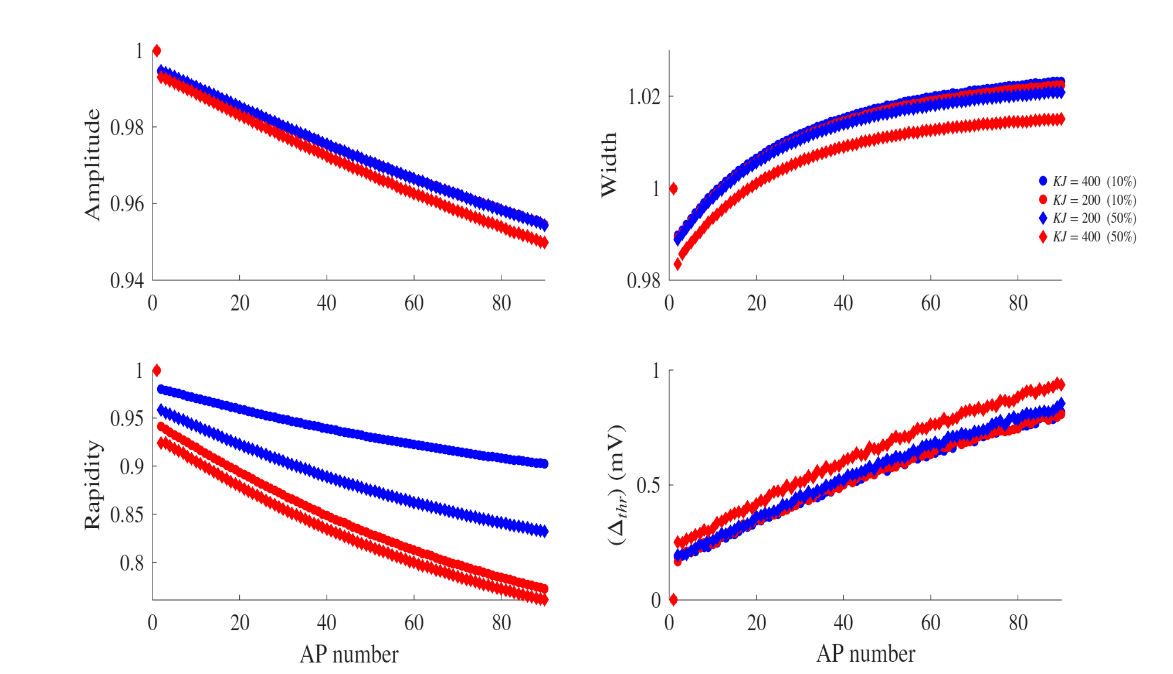


**Figure S2: Impact of coupling strength (KJ) and fraction of cooperative gating (p) on the magnitude of AP variation.** Circles represent values where the fraction of cooperatively activating Na^+^ channels (p) is 10%, and diamond symbols represent values where the fraction is 50% (i.e., half of the Na^+^ channels activate cooperatively). The color of the symbol reflects the coupling strength (KJ): blue indicates weak coupling (KJ = 200mV), and red indicates strong coupling KJ =400mV).


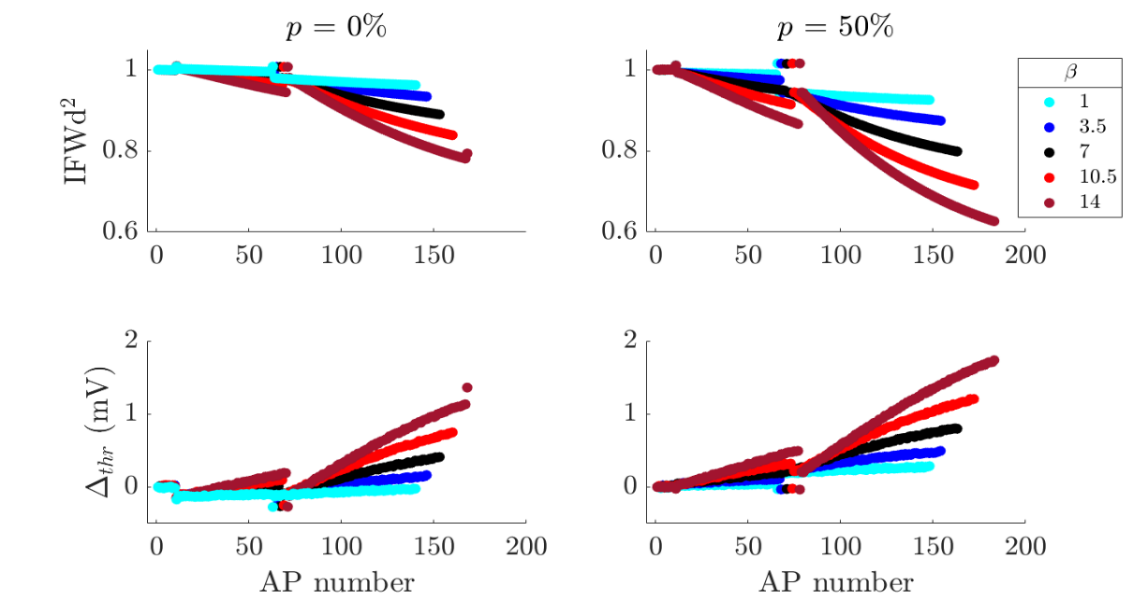


**Figure S3:** **Impact of the intracellular to extracellular volume ratio on AP threshold and rapidity.** The AP rapidity is normalized to the rapidity of the first spike and $\Delta_{thr}=Threshold-Threshold_{1st}$. Left: the values with no cooperative Na^+^ channels. Right: the values with 50% cooperative Na^+^ channels (KJ = 400 mV). The results were obtained with 3 steps current and intra sweep interval of 5 s.


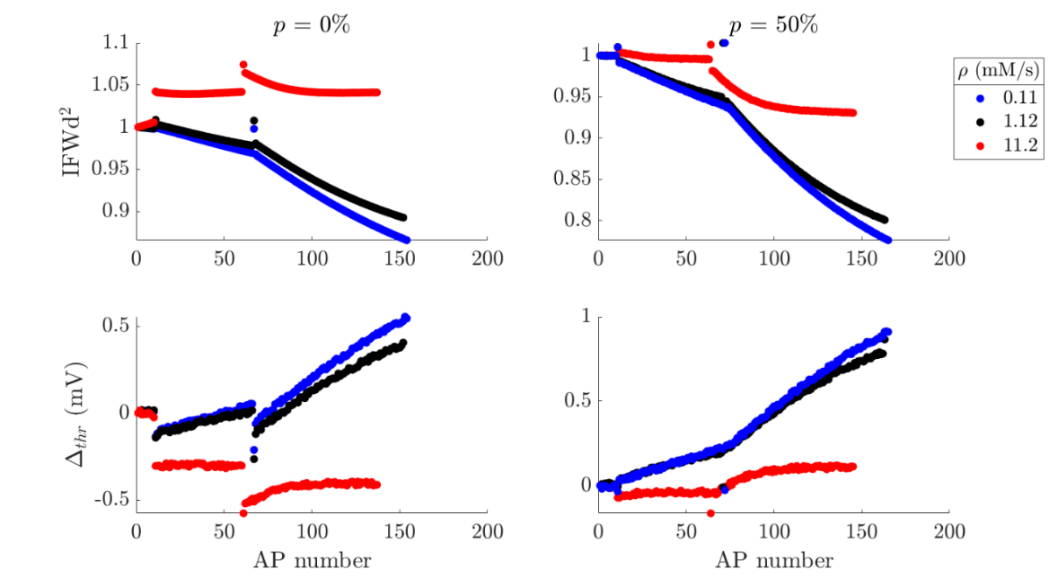


**Figure S4**: **Impact of the Na/K pump strength on AP threshold and rapidity.** Left: the values with no cooperative Na^+^ channels. Right: the values with 50% cooperative Na^+^ channels (KJ = 400 mV). The results were obtained with 3 steps in current and inter-pulse intervals of 5 s. Note that the scale is not the same between independent and cooperative gating to show the difference in all three cases.


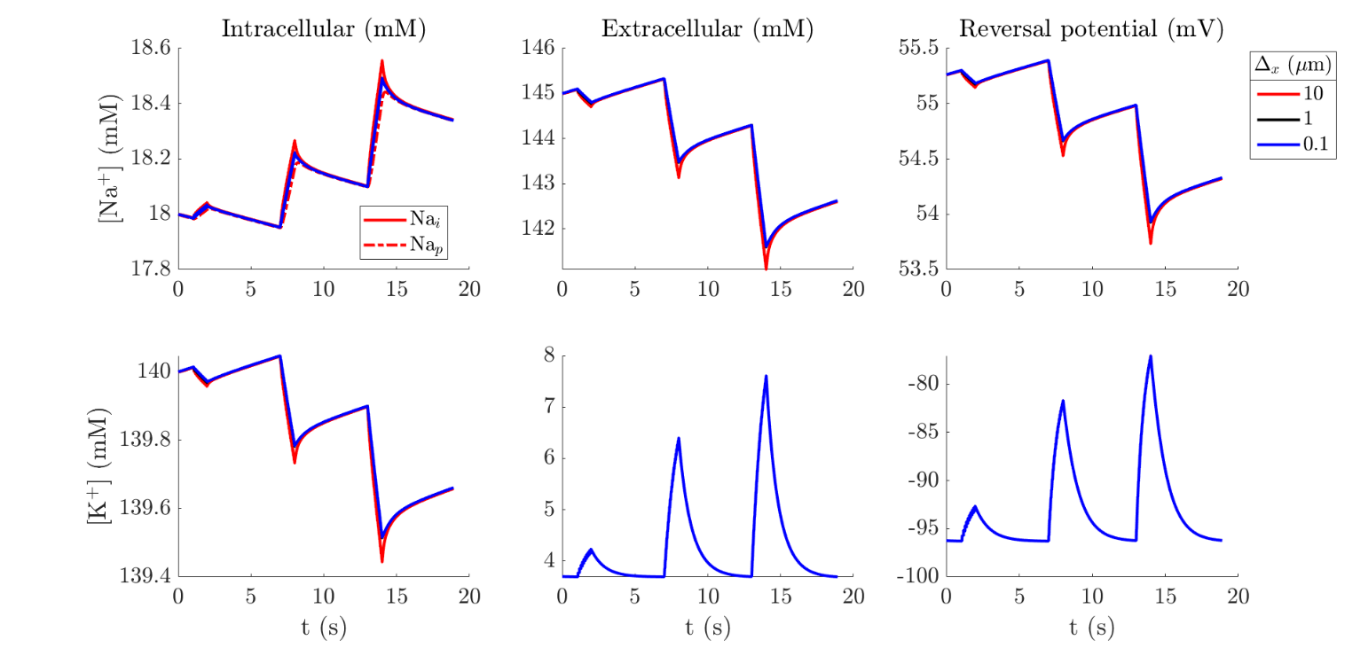


**Figure S5:** Impact of spacing distance between the pump and Na^+^ channels on ion concentrations and reversal potential ($D_{Na}$ = 0.3 um^2^/ms).


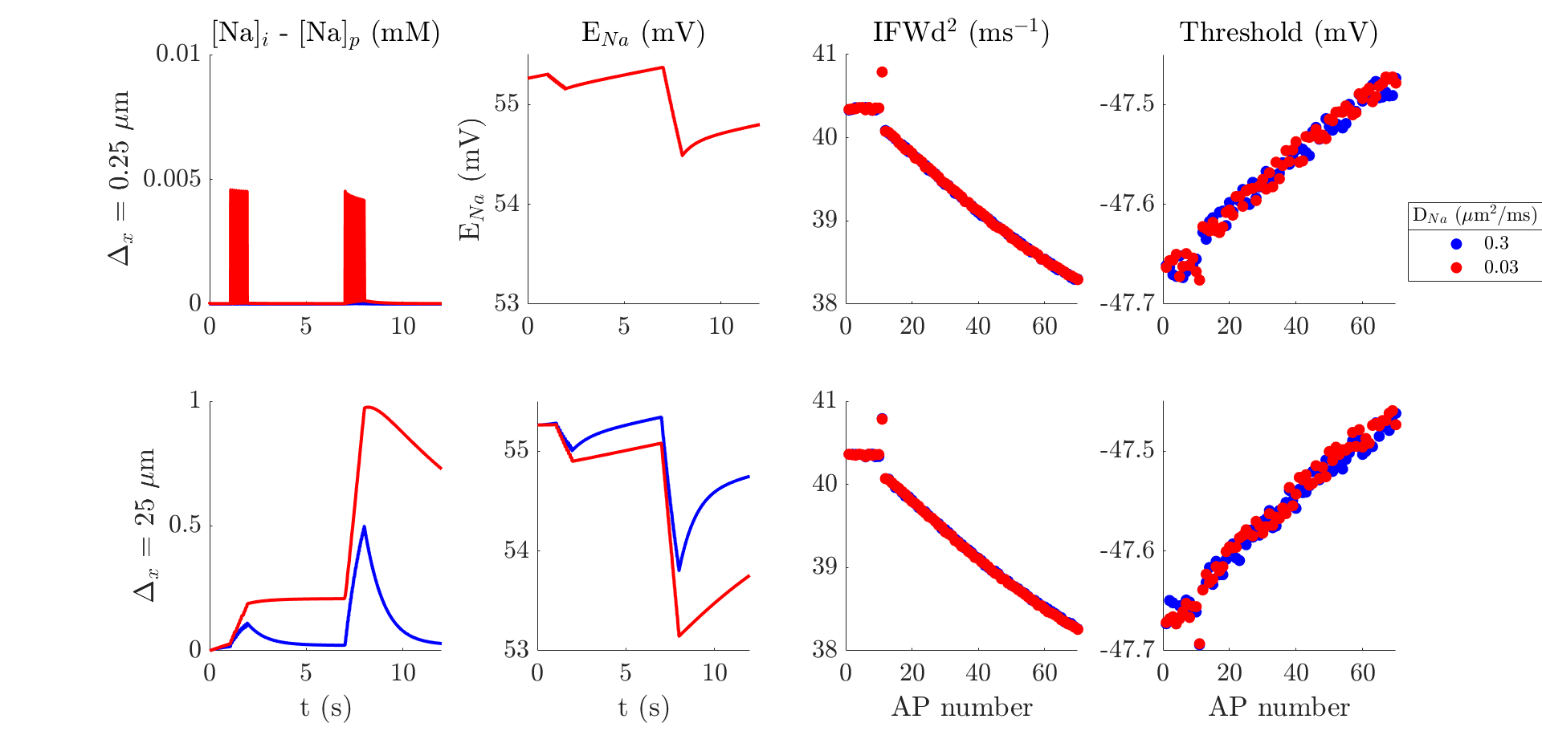


**Figure S6:** Impact of spacing difference between the pump and Na+ channels and diffusion coefficient on concentration with cooperative gating (p =0.5, KJ =400 mV). Na^+^ concentration difference between the pump and Na^+^ channels (first column), its effect on Na^+^ channel reversal potential (second column), AP rapidity (third column), and AP threshold potential (fourth column). Blue traces and circles indicate the value when using the reported diffusion coefficient ($D_{Na}=0.3$ µm^2^/ms). red traces and circles indicate the value when using slow diffusion coefficient ($D_{Na}=0.03$ µm^2^/ms). The spacing between the channels and the pump was 0.25 µm (top row), or 25 µm (bottom row).


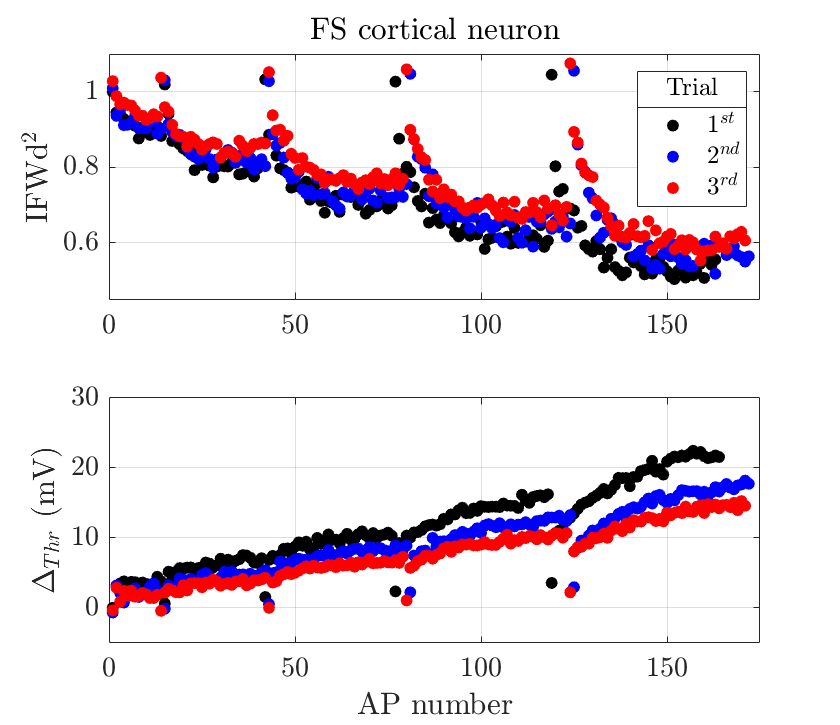


**Figure S7:** Normalized AP rapidity (top) and threshold potential difference from the value of the first AP. The step-and-hold stimulation protocol included 5 steps of 0.5s long depolarization pulses with an inter-sweep interval of 6.5 s. The stimulus train was repeated 3 times with a 20 s interval. The recordings obtained from the GigaScience database [41],
